# Biomineralized hybrid nanodots for tumor therapy *via* NIR-II fluorescence and photothermal imaging

**DOI:** 10.3389/fbioe.2022.1052014

**Published:** 2022-10-31

**Authors:** Xuegang Niu, Penghui Wei, Jiangnan Sun, Yuanxiang Lin, Xiaoyong Chen, Chenyu Ding, Yang Zhu, Dezhi Kang

**Affiliations:** ^1^ Department of Neurosurgery, Neurosurgery Research Institute, The First Affiliated Hospital of Fujian Medical University, Fuzhou, China; ^2^ Department of Psychology, The First Affiliated Hospital of Fujian Medical University, Fuzhou, China; ^3^ Department of Chemistry, University of Science and Technology of China, Hefei, China

**Keywords:** chemodynamic therapy, NIR-II fluorescence imaging, reactive oxygen species, photodynamic therapy, tumor

## Abstract

Chemodynamic therapy (CDT) is an emerging and promising therapeutic strategy that suppresses tumor growth by catalytically converting intracellular hydrogen peroxide (H_2_O_2_) into highly-reactive hydroxyl radicals (•OH). However, the inherent substrate of H_2_O_2_ is relatively insufficient to achieve desirable CDT efficacy. Therefore, searching for integrated therapeutic methods with synergistic therapeutic modality is especially vital to augment therapeutic outcomes. Herein, we reported nanodot- Cu_x_Mn_y_S_z_ @BSA@ICG (denoted as CMS@B@I) and bovine serum albumin (BSA)-based biomineralization Cu_x_Mn_y_S_z_ (CMS) loaded with photodynamic agent-indocyanine green (ICG). CMS@B@I converts endogenous hydrogen peroxide (H_2_O_2_) into highly active hydroxyl radical (•OH) via Fenton reaction, and effectively produces reactive oxygen species (ROS) after being exposed to 808 nm laser irradiation, attributable to the excellent photodynamic agent-ICG. This results in eliciting a ROS storm. Additionally, CMS@B@I exhibits a superior photothermal effect under NIR-II 1064 nm laser irradiation to enhance tumor CDT efficacy. The NIR-II fluorescence imaging agent of ICG and the excellent photothermal effect of CMS@B@I are highly beneficial to NIR-II fluorescence and infrared thermal imaging, respectively, resulting in tracing the fate of CMS@B@I. This study attempts to design a bimodal imaging-guided and photothermal-enhanced CDT nanoagent for augmenting tumor catalytic therapy.

## Introduction

Chemodynamic therapy (CDT) is an emerging and promising therapeutic strategy that suppresses tumor growth *in situ* by converting hydrogen peroxide (H_2_O_2_) into a highly-toxic hydroxyl radical (•OH) via Fenton or Fenton-like reactions (Zhang et al., 2016b; Lin et al., 2018; Ma et al., 2018; Yang B. et al., 2019; Fan et al., 2019; Lin et al., 2019; Min et al., 2019; Wang et al., 2019; Lin et al., 2020; Sang et al., 2020; Zhu et al., 2021b; Cheng et al., 2021; Fu et al., 2021; Wang et al., 2021). The tumor microenvironment (TME) comprises hypoxia (Chen et al., 2017; Dai et al., 2020), weak acidity (Gatenby and Gillies, 2004; Zhu et al., 2022a), and H_2_O_2_ overproduction owing to the excessive metabolism of cancer cells (Dai et al., 2020; Chen et al., 2022). To date, several nanomaterials with TME stimuli-responsive Fenton or Fenton-like activity have been developed for the CDT of tumors by disrupting intracellular redox homeostasis (Zhang et al., 2016a; Dong et al., 2018; Yang Y. et al., 2019; Gao et al., 2019; Liu et al., 2019; Ma et al., 2019; Zhu et al., 2022a). Iron, manganese, and copper-based nanomaterials with an excellent photothermal effect have indicated profound catalytic performance and impressive antitumor chemodynamic efficacy (Liu et al., 2015; Chang et al., 2019; Cao et al., 2021). However, due to the high level of GSH in the tumor microenvironment, the undesired ability to scavenge reactive oxygen species (ROS) significantly reduced the CDT efficacy (Lyu et al., 2020; Xiong et al., 2021). Mono-modal therapy has been investigated and resulted in limited treatment efficacy. Therefore, exploring novel integrated therapeutic methods with synergistic therapeutic modality is particularly crucial to augment therapeutic outcomes (Feng et al., 2019; Zhao et al., 2020).

Copper sulfide (CuS) nanoparticles have been widely utilized for phototherapy therapy (PTT), owing to their intrinsic near-infrared (NIR) absorption (Huang et al., 2014; Wang et al., 2016). Recently, manganese decorated CuS nanoparticles have demonstrated a promising outcome as theranostic nanomedicine for multimodal imaging guided PTT of tumors (Liu et al., 2015; Ke et al., 2017). However, the intrinsic existence of univalent elements (Cu and Mn) and critical preparation conditions have limited a desirable CDT effect. We anticipate that multivalent nanomaterials with excellent catalytic activity can achieve selective catalytic therapy in response to TME (Liu et al., 2020), which exhibited negligible side effects on normal tissue.

Biomineralization is a common process used to form biomaterials by combining biomacromolecules (protein and nucleic acid) with metal or inorganic ions under physiological conditions (Wang et al., 2020). In recent years, biomineralization encouraged the fabrication of various nanoparticles and has been studied for the diagnosis and therapy of tumors (Jiang et al., 2022). For instance, Zhu et al. reported that ferritin-based biomineralization fabricates MnO_2_ nanozymes with superior stability, and possesses a small uniform size for enhanced photodynamic therapy, which shows limited systemic cytotoxicity and excellent biodegradability. Therefore, the rational design-based biomineralization of nanoparticles could effectively overcome the drawbacks of traditional strategies.

**SCHEME 1:**
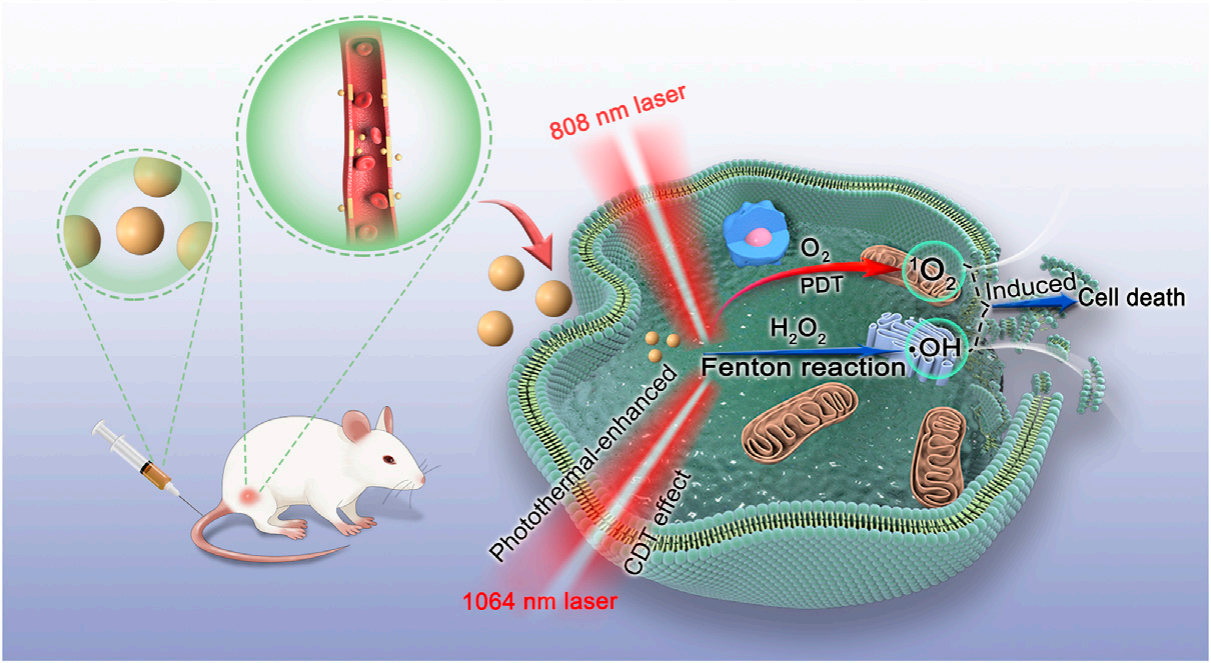
Schematic illustration of the mechanism of CMS@B@I for PDT and photothermal-enhanced CDT effects.

Herein, bovine serum albumin (BSA)-based biomineralization was employed to construct NIR-II fluorescence and photothermal imaging-guided nanodot-Cu_x_Mn_y_S_z_@BSA@ICG (denoted as CMS@B@I) for PDT and photothermal enhanced chemodynamic therapy. The intrinsic existence of multivalent elements (Mn and Cu) endow CMS@B@I with profound catalytic performance, which was used to convert overproduced H_2_O_2_ into highly reactive •OH (Scheme 1). Meanwhile, CMS@B@I also exhibited promising PDT results after being exposed to an 808 nm laser, owing to the PDT agent-indocyanine green (ICG), resulting in an ROS storm in tumor cells. Additionally, CMS@B@I demonstrated excellent photothermal conversion efficiency (PCE, ƞ = 35.3) under NIR-II 1064 nm laser irradiation for augmenting CDT efficacy. Taking advantage of these properties, CMS@B@I was also performed on NIR-II fluorescence and photothermal imaging. Furthermore, both *in vitro* and *in vivo* experimental results demonstrated that CMS@B@I can significantly inhibit tumor growth with negligible side effects in mice under laser irradiation. This study paves the way for designing imaging-guided and photothermal-enhanced CDT for cancer.

## Materials and methods

### Materials

Analytical grade copper chloride (CuCl_2_.2H_2_O), ascorbic acid (AA), sodium hydroxide (NaOH), manganese chloride (MnCl_2_), and thioacetamide were obtained from Sinopharm Chemical Reagents (Shanghai, China). BisBenzimide H 33342 trihydrochloride (Hoechst 33342), 3, 3′, 5, 5′-Tetramethylbenzidine (TMB), 1,3-diphenylisobenzofuran (DPBF), 2′,7′-dichlorofluorescin diacetate (DCFH-DA), and a JC-1 kit were purchased from Beyotime (Shanghai, China). Dulbecco’s modified eagle medium, fetal bovine serum (FBS), penicillin-streptomycin, and 0.25% trypsin-EDTA were supplied by Hyclone (Logan, United States). 5-Tert-Butoxycarbonyl-5-methyl- 1-pyrroline N-oxide (BMPO) and 5,5-dimethyl-1-pyrroline n-oxide (DMPO) were acquired from Dojindo (Dojindo, China). Other reagents were bought from Sigma-Aldrich (St. Louis, United States) or Sinopharm Chemical Reagent (Shanghai, China). Deionized (DI) water was obtained from a Milli-Q Water Purification system (Millipore, Billerica, MA).

### Synthesis of CMS@B nanodots

CuCl_2_·H_2_O (50 mg), MnCl_2_⋅4H_2_O (50 mg) and BSA (200 mg) were dispersed in 100 ml of DI water during drastic magnetic stirring for 10 min. Then, NaOH (1 M, 10 ml) and ascorbic acid (1 M, 10 ml) were added to the solution successively during magnetic stirring. After 10min, 1 ml 0.1 M Na_2_S was added and the temperature was maintained at 90°C for 1 h. Then, CMS@B was placed into a 3 k dialysis bag and dialyzed for 48 h for subsequent use.

### Synthesis of CMS@B@I

The above-derived CMS@B nanodots were dispersed in 20 ml DI water, and 5 ml 1 mg/ml ICG was added during drastic magnetic stirring. After 12 h, the solution was placed into a 1 k dialysis bag and dialyzed for 48 h and freeze-dried for subsequent use.

### Measurement of fenton activity

The catalytic activity of HNC-supported CMS@B was measured by using TMB as a probe, which can be converted into oxidized blue TMB by •OH. HNC-supported CMS@B (100 μg/ml) and H_2_O_2_ (1 mM) were successively introduced into the TMB (1 mM) solutions, and the mixtures were shaken at 37°C for 10 min. After centrifugation, the absorption spectra of the supernatant was measured.

A terephthalic acid photoluminescence assay was employed to detect the generation of hydroxyl radicals. 4 mM H_2_O_2_, 0.5 mM terephthalic acid and CMS@B@I (200 μg/ml) were incubated in a 0.01 M HAc-NaAc buffer (pH 5) at 37°C for various durations. The mixture was measured by a fluorescence spectrophotometer at 410 nm.

### Electron paramagnetic resonance detect the hydroxyl radical

DPBF employed trap •O_2_^−^ generated by CMS@B@I. In a typical assay, 200 μg/ml CMS@B@I, 4 mM H_2_O_2_ and 25 μg/ml DPBF were incubated in a 0.01 M HAc-NaAc buffer (pH 5.0) at 25°C. Then, the reaction was detected by a UV-vis spectrophotometer at 420 nm, for different time points.

### Calculation of the photothermal conversion efficiency

To compute the photothermal conversion efficiency of CMS@B@I, the temperature of the solution under 1064 nm laser irradiation was recorded and calculated according to the equation described. CMS@B@I (1 ml 600 μg/ml) was introduced in a glass vial and irradiated by a 1,064 nm laser (1 W/cm^2^) for 600 s. The laser was then turned off until it cooled to room temperature. The temperature of CMS@B@I was monitored by a thermocouple microprobe submerged in the solution. The experiment was repeated five times to investigate the photothermal stability of the material.

### Cell culture and animals

4T1 cells were bought from Shanghai Institute of Biochemistry and Cell Biology (Shanghai, China). 18–20 g of BALB/c mice (female) were from Beijing HFK Bioscience Co., Ltd. All animal protocols were approved by the Ethical Committee of Fujian Medical University. Pentobarbital sodium was used to anesthetize (50 mg/kg) and euthanize (150 mg/kg) the mice *via* intraperitoneal injection. For 4T1 murine breast tumor model, 4T1 tumors were exo-grafted through subcutaneous injection of 100 μl phosphate-buffered saline of 4T1 cells (1 × 10^6^ cells) in the flank region of the back. The tumor-bearing mice were then used for subsequent photothermal imaging *in vivo* and antitumor treatment until the tumor volume reached 70 mm^3^. The tumor volume was defined as (tumor length) × (tumor width)^2^/2.

### Cellular uptake

4T1 cells were seeded in confocal dishes at a density of 2 × 10^5^ cells per dish and cultured for 24 h. After replacing the medium with 1 ml of the CMS@B@I (100 μg ml^−1^), the cells were further incubated at various durations (0, 20, 40, and 60 min). Next, the medium was removed and the 1.50 ml DMEM containing 2 μl Hoechst 33342 (10 mg/ml) was added. After 20-min incubation, the medium was removed and the cells were washed with PBS three times. The fluorescence imaging of the cells was analyzed *via* confocal microscopy.

### Cytotoxicity and cell apoptosis assessments

The cell viability was measured by a methyl thiazolyl tetrazolium (MTT) assay and live/dead cell staining assay. For an MTT assay, 4T1 cells were seeded in 96-well plates at 6,000 cells per well and cultured for 12 h. The cells were treated with several concentrations of CMS@B@I, and further divided into four groups. One group was irradiated with 808 nm laser, one was irradiated with 1064 nm laser, one was irradiated with 808 and 1,065 nm lasers, and the final group was not irradiated. After incubation for 4 h to ensure that the material was internalized by the cells, the irradiation group was exposed to a laser for 5 min. After further incubation for 20 h, the medium was replaced with a fresh neutral culture medium and 10 μl 10 mg/ml MTT solution was added. The cells were incubated for another 4 h to reduce the yellow MTT into dark blue formazan crystals. Finally, the formazan product was dissolved in dimethyl sulfoxide (DMSO) and quantified by absorbance at 570 nm using a Bio-Rad 680 microplate reader.

To analyze cell apoptosis, Annexin V-FITC and PI assays were employed. 4T1 cells were seeded in 6-well plates at a density of 6 × 10^5^ cells per well and incubated for 24 h. Subsequently, 1 ml 200 μg/ml CMS@B@I was added and incubated for 4 h. The cells treated were divided into four groups. One group was irradiated with an 808 nm laser, one was irradiated with a 1,064 nm laser, one was irradiated with 808 nm and 1,065 nm lasers, and the final one was not irradiated. After another 20 h, the cells were washed three times and harvested for treatment with Annexin V-FITC and PI according to manufacturer’s instructions. The quantitative apoptotic assay was gauged using flow cytometric analysis.

### ROS detection in cells

The ROS level in 4T1 cells was detected with a DCFH-DA Kit. 4T1 cells were seeded in 6-well plates and cultured for 12 h. After incubation at 37°C in 5% CO_2_ for 24 h, the cells were treated with 1 ml 200 μg/ml CMS@B@I and incubated for another 12 h. The irradiation group was exposed to a 1,064 nm laser with 1 W/cm^2^ or an 808 nm laser with 0.3 W/cm^2^ for 5 min. Next, the 1.5 ml medium was removed and the DMEM containing DCFH-DA (10 μM) and 2 μl Hoechst 33342 (10 mg/ml) was added. After 20 min of incubation, the medium was removed and the cells were washed with PBS three times. The fluorescence imaging of the cells was analyzed by fluorescence microscopy.

### Analysis of the change of mitochondrial membrane potential

JC-1 is a fluorescence probe that targets mitochondria and emits red fluorescence from healthy mitochondrial membranes that exhibit high potential (fluorescence corresponds JC-1 aggregates). Conversely, JC-1 emits green fluorescence when it accumulates in disrupted mitochondrial membranes with low potential (JC-1 monomers). To investigate the mitochondrial membrane potential, 4T1 cells were seeded in a confocal dish and incubated for 12 h. Subsequently, the DMEM was replaced with fresh acidified DMEM comprising CMS@B@I for 4 h of incubation. The treated cells were divided into four groups. One group was irradiated with an 808 nm laser, one was irradiated with a 1,064 nm laser, one was irradiated with 808 nm and 1,065 nm lasers, and the final one was not irradiated. After another 20 h, the cells were washed with PBS three times and treated according to the JC-1 kit. The fluorescence imaging of the cells was analyzed by confocal microscopy.

### *In vivo* infrared thermal imaging

The thermal signal was obtained using an infrared thermal camera (Fotric 225S). Balb/c mice with 4T1 tumors were i. v. Injected with CMS@B@I (200 μl, 3.0 mg/ml). After 24 h, the tumors were exposed to a laser (1.0 W/cm^2^, 1,064 nm) for 5 min. The figures were analyzed by the AnalyzIR software.

### *In vivo* antitumor assay

4T1 tumor-bearing mice were randomly divided into six groups (five mice per group) and treated under varying conditions: I (PBS), II (PBS plus L), III (CMS@B@I), IV (CMS@B@I plus 808 nm laser), V (CMS@B@I plus 1,064 nm laser), and VI (CMS@B@I plus 808 and 1,064 nm lasers). The mice in the CMS@B@I were given a dose of 3 mg/kg via intravenous tail injection. After 24-h post-injection, the 808 nm and 1,064 nm laser groups were exposed to a laser (0.3 W/cm^2^, 808 nm or 1.0 W/cm^2^, 1,064 nm) for 5 min. Nanoparticle administration and irradiation were performed only once for antitumor treatment. The tumor size and weight were measured and recorded every other day by using digital calipers and an electronic balance. After a 14-day treatment, the mice were euthanatized, and their tumor tissues were harvested and weighed. After histological analysis, major organs (the heart, lungs, liver, spleen, and kidneys) were collected, and tumors were observed with hematoxylin and eosin (H&E) stained pathological sections. The tumors were used in hypoxia and anti-angiogenesis immunofluorescence assay.

### Statistical analysis

All quantitative data are expressed as the mean ± standard deviation (SD). Differences between the two groups were inspected using the Student’s two-tailed *t*-test, and a comparison of multiple groups was performed using one way analysis of variance (ANOVA). The results were considered significant at *p < 0.05, **p < 0.01, ***p < 0.001, n ≥ 3.

## Results and discussion

**FIGURE 1:**
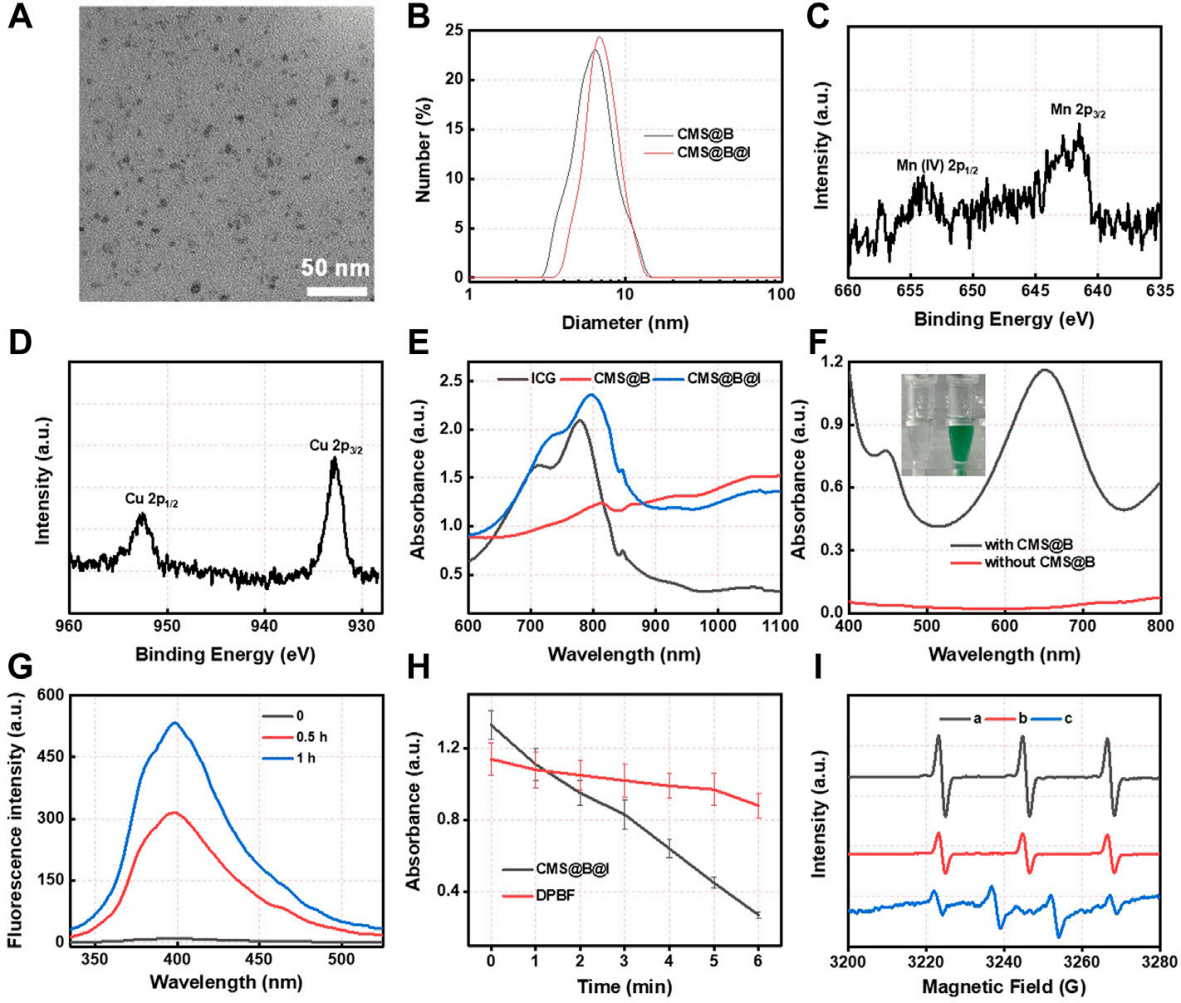
**(A)** TEM images of CMS@B@I. **(B)** DLS size distribution of various nanoparticles. **(C)** High resolution Mn 2p XPS spectrum. **(D)** High resolution Cu 2p XPS spectrum. **(E)** UV-vis spectra of different nanoparticles and ICG. **(F)** UV-vis spectra of TMB (1 mM) incubated with H_2_O_2_ (1 mM) in the presence of CMS@B. **(G)** UV-vis spectra of Ti(SO_4_)_2_ incubated with CMS@B@I-pretreated H_2_O_2_. **(H)** The detection of superoxide radical by using DPBF kit. **(I)** The ESR spectrum detect the •OH and ^1^O_2_ by using BMPO and TEMP as a trapping agent, respectively. **(A)** TEMP + CMS@B@I + laser, **(B)** TEMP + ICG + laser, and **(C)** BMPO + CMS@B@I + H_2_O_2_. *p < 0.05, **p < 0.01, ***p < 0.001.

The biomineralization was conducted to prepare CMS@B@I using BSA as a stabilizer. As displayed in Figure 1A, transmission electron microscopy (TEM) revealed spherical CMS@B@I with a uniform size of ∼5 nm. The hydrodynamic diameter and zeta potential were measured using dynamic light scattering (DLS). As shown in Figure 1B and Supplementary Figure S1, the hydrodynamic diameter of CMS@B@I was about 10 nm, and the zeta potential was −27 mV. X-ray photoelectron spectroscopy (XPS) was employed to investigate the valence states of Mn and Cu species in CMS@B@I (Figures 1C,D and Supplementary Figure S2). According to XPS spectrum, Cu 2p in CMS@B@I can be divided into two peaks, namely, Cu^+^ and Cu^2+^. Moreover, Mn 2p in CMS@B@I can also be divided into two peaks, namely, Mn^2+^ and Mn^4+^. The multivalent elements of Cu and Mn can endow CMS@B@I with profound Fenton activity (Lin et al., 2019; Zhu et al., 2021b). In addition, the ultraviolet-visible (UV-vis) spectrum indicated that the PDT agent-ICG was successfully loaded in CMS@B (Figure 1E). Moreover, the stability of CMS@B@I in DMEM was performed by DLS (Supplementary Figure S3).

The Fenton activity of CMS@B was investigated using 3,3′,5,5′-tetramethylbenzidine (TMB) as a probe (Figure 1F). The results demonstrate that CMS@B could efficiently oxidize the TMB at an acidic pH in response to TME, owing to the multivalent elements of Mn and Cu. Meanwhile, the fluorescence spectra of terephthalate acid was oxidized by CMS@B@I in the presence of H_2_O_2_. The results demonstrate that CMS@B@I can successfully convert H_2_O_2_ into highly active •OH, which can oxidize terephthalate acid to generate a strong fluorescence intensity (Figure 1G). We also performed 1,3-diphenylisobenzonfuran (DPBF) as a •OH trapping agent to evaluate the •OH production ability of CMS@B@I in the presence of H_2_O_2_ (Figure 1H and Supplementary Figure S4). Electron spin resonance (ESR) was employed to investigate the catalytic mechanism of CMS@B@I using 5-tertbutoxycarbonyl-5-methyl-1-pyrroline N-oxide (BMPO) and 2.2,6,6-tetramethylpiperidine (TEMP) as the specific spin trap reagents (Figure 1I). Triplet characteristic peaks 1:1:1 of TEMP-^1^O_2_ confirmed the production of ^1^O_2_ under 808 nm laser irradiation; while quadruple characteristic peaks 1:2:2:1 of BMPO-•OH confirmed the production of •OH. These results indicate that CMS@B@I can effectively produce •OH and ^1^O_2_ exposed to an 808 nm laser, demonstrating the excellent CDT effect and PDT of CMS@B@I.

**FIGURE 2:**
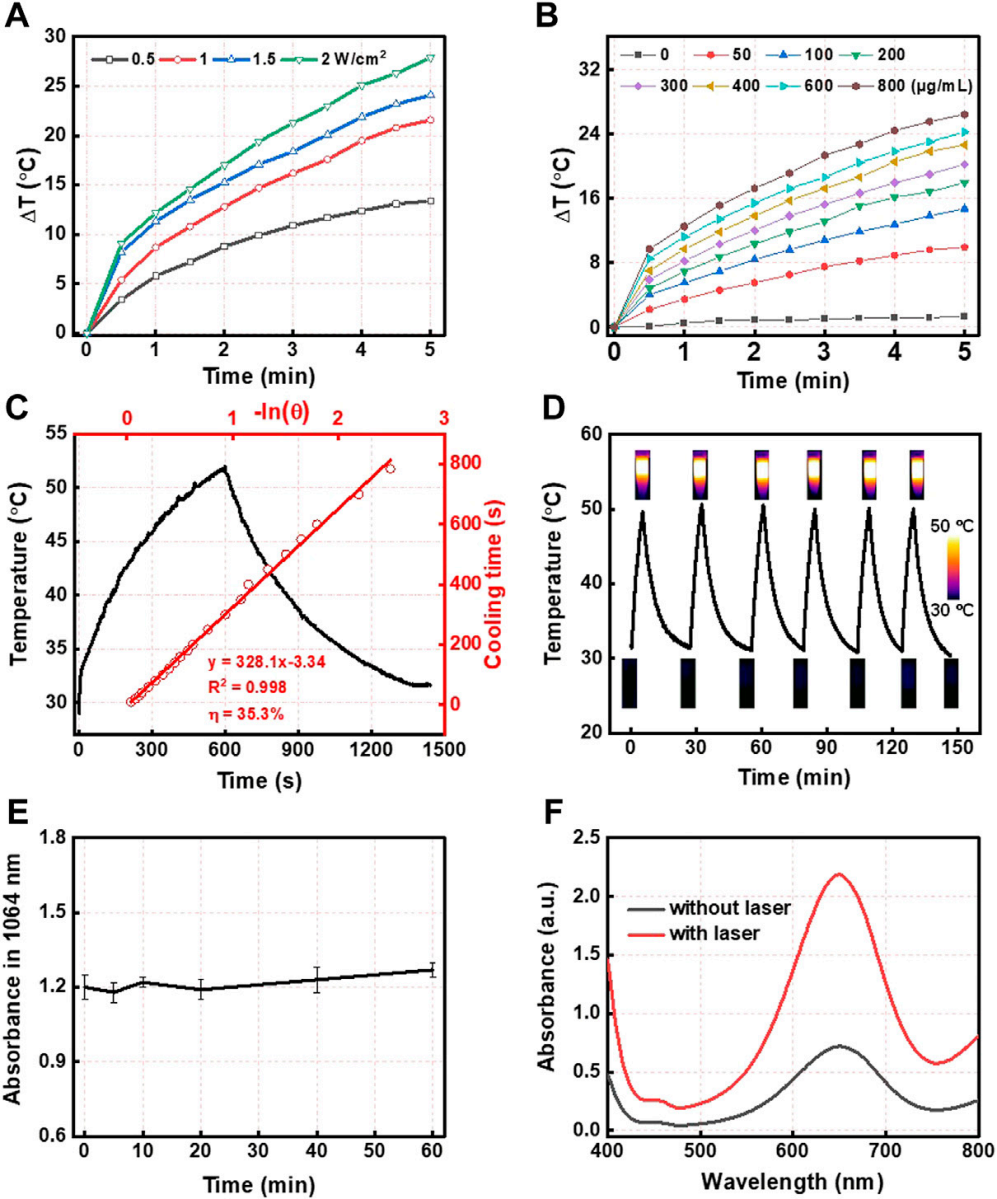
**(A)** The photothermal effect of different powers and **(B)** different concentrations of CMS@B@I under 1,064 nm laser irradiation. **(C)** Calculation of the photothermal conversion efficiency. **(D)** Temperature change profiles of CMS@B@I in water with repeating irradiation cycles with 1,064 nm laser. **(E)** The photothermal stability of CMS@B@I under 1,064 nm lasers irradiation. **(F)** UV-vis spectra of TMB (1 mM) incubated with H_2_O_2_ (1 mM) in the presence or absence of 1,064 nm lasers irradiation.

**FIGURE 3:**
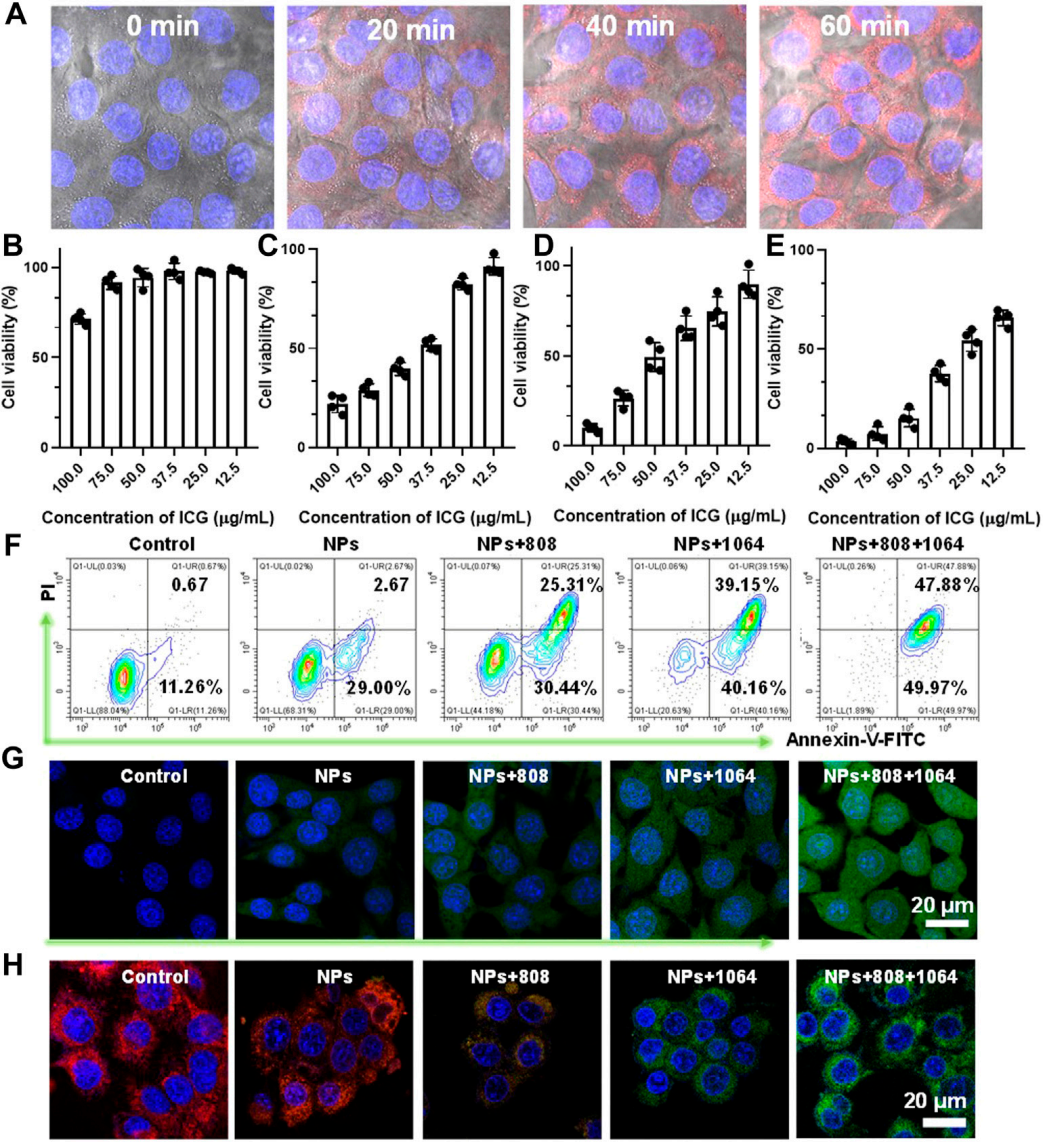
**(A)**The cellular uptake behavior of CMS@B@I. **(B)** Viability of 4T1 cells treated with different concentrations of CMS@B@I. **(C)** Viability of 4T1 cells treated with different concentrations of CMS@B@I plus 808 nm laser. **(D)** Viability of 4T1 cells treated with different concentrations of CMS@B@I plus 1,064 nm laser. **(E)** Viability of 4T1 cells treated with different concentrations of CMS@B@I plus 808 nm and 1,064 nm laser. **(F)** Flow cytometry analyzed the apoptosis rate after different treatments. **(G)** DCFH-DA stained 4T1 cells treated with CMS@B@I under 808 nm or 1,064 nm laser irradiation for 5 min **(H)** CLSM images analyzed mitochondrial depolarization by using JC-1 kit. *p < 0.05, **p < 0.01, ***p < 0.001.

Furthermore, based on the wide absorption of CMS@B@I in the NIR I and II bio-window (Figure 1E), CMS@B@I exhibited impressive photothermal performance in a concentration-dependent manner under a 1,064 nm laser (Figures 2A,B). Compared with the NIR I bio-window (750–1,000 nm), the NIR II bio-window (1,000–1,400 nm) has attracted increasing attention due to its deeper tissue penetration depth and higher maximum permissible exposure to the laser (1 W/cm^2^) than that of 808 nm (0.33 W/cm^2^) (Feng et al., 2019; Cui et al., 2022). The photothermal conversion efficiency (PCE) of the CMS@B@I was measured by irradiation with a 1,064 nm laser with 1.0 W/cm^2^, from 0 to 10 min (Figure 2C). The temperature of CMS@B@I was monitored using an infrared thermal camera (Fotric 225S), and the PCE was calculated to be *η* = 35.3%, which was higher compared to that of Au nanorods (21%), Cu_2x_Se nanocrystals (22%), and BP quantum dots (28%) (Hessel et al., 2011; Zeng et al., 2013; Sun et al., 2015). Furthermore, CMS@B@I exhibited excellent photothermal stability, demonstrating its viability as a photothermal agent (Figures 2D,E). Inspired by the excellent photothermal effect of CMS@B@I, we performed catalytic performance after irradiation. The results demonstrate that CMS@B@I has higher Fenton activity compared to the absence of laser irradiation (Figure 2F). These results demonstrated that CMS@B@I exhibited enhanced catalytic activity after irradiation. Additionally, infrared thermal imaging demonstrated that the temperature of various time periods was increased (Figure 3D). The above results demonstrate that CMS@B@I is a viable multimodal imaging-guided photothermal agent for boosting the CDT effect.

**FIGURE 4:**
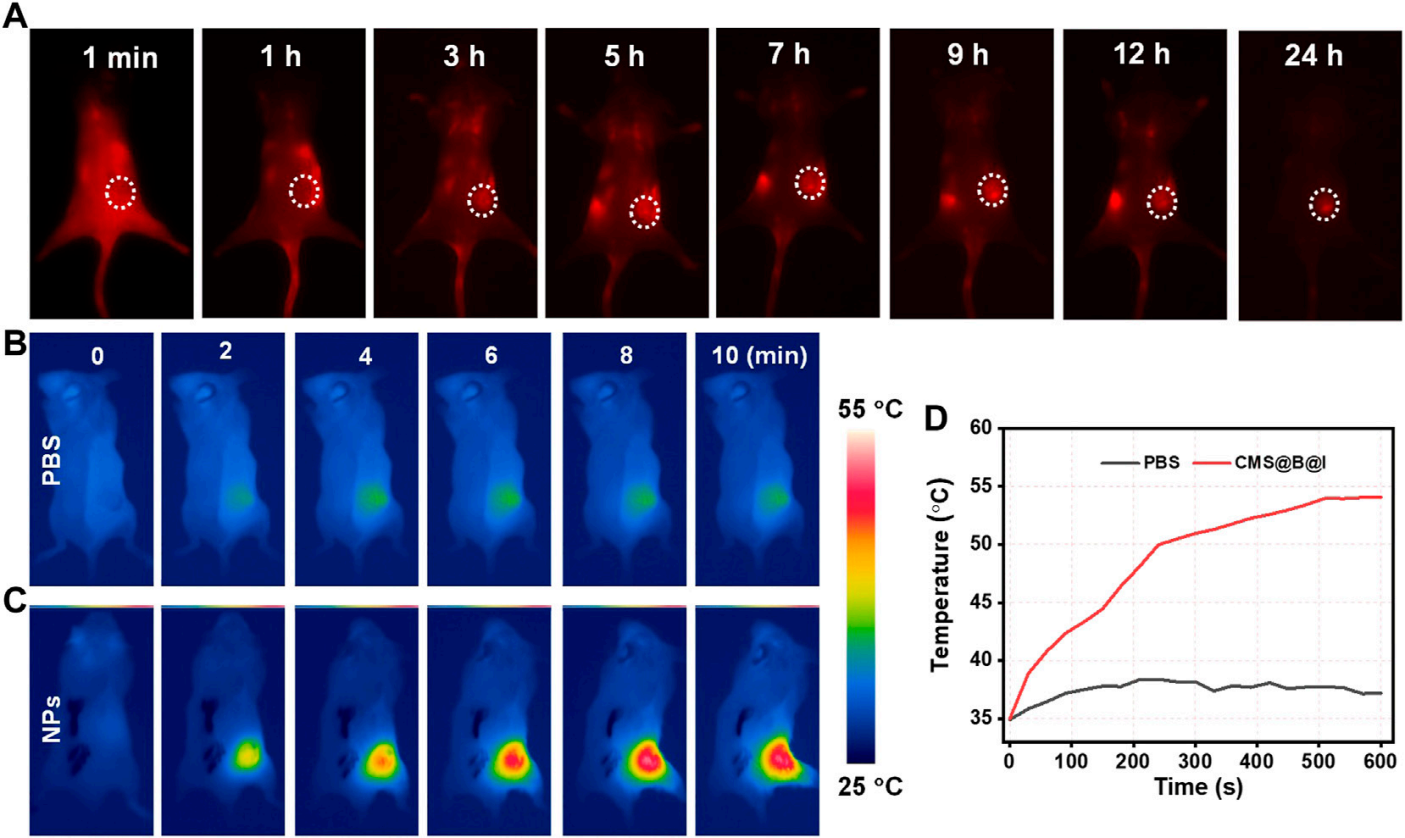
**(A)** The NIR-II fluorescence images of tumor in 4T1 tumor-bearing mice at different time points. **(B)** THE infrared thermal images of PBS and **(C)** CMS@B@I of tumor in 4T1 tumor-bearing mice at different time points. **(D)** The corresponding temperature change curves of the tumor in 4T1 tumor-bearing mice.

The cellular uptake behavior and *in vitro* therapeutic effect of CMS@B@I were evaluated. Firstly, the red fluorescence signal of ICG in 4T1 cells was monitored by confocal laser scanning microscopy (CLSM). According to Figure 3A and Supplementary Figure S5, the red fluorescence of CMS@B@I gradually increased in a time-dependent manner, verifying that CMS@B@I can be efficiently internalized via endocytosis. Encouraged by the photothermal-enhanced catalytic activity and remarkable PDT effect, a cytotoxicity assay of CMS@B@I was performed on 4T1 cells using a 3-(4,5-dimethylthiazol-2-yl)-2,5-diphenyltetrazolium bromide (MTT) assay. As predicted, CMS@B@I (ICG: 100 μg/ml) group exhibited higher cytotoxicity on tumor cells, which can be ascribed to the abundant **·**OH production via the Fenton reaction (Figure 4B and Supplementary Figure S6). Additionally, about 75% of the 4T1 cells were killed after being further exposed to an 808 nm laser (0.3 W/cm^2^, 5 min), 90% of cancer cells were killed after being exposed to 1,064 nm laser (1.0 W/cm^2^, 5 min), and almost all tumor cells were killed after being exposed to both 808 and 1,064 nm lasers. This is attributable to the PDT efficacy and photothermal-amplified catalytic therapy. Flow cytometric analysis was also performed to support the cell growth inhibition of CMS@B@I (Figure 3F). It is distinct that CMS@B@I induced 31.67% cell apoptosis by triggering the Fenton activity, while a remarkable increasing cell apoptosis effect emerged after being combined with PTT and PDT effects. The catalytic products of cellular OH and ^1^O_2_ by the Fenton reactions and PDT effect were evaluated using a ROS probe 2,7-dichlorofluorescin diacetate (DCFH-DA). The results revealed that CMS@B@I could efficiently increase the cellular ROS level. Moreover, the laser irradiation further elevated the production efficacy of ROS; this is consistent with the results of photothermal-enhanced Fenton activity (Figure 3G and Supplementary Figure S7). The probe 5.5′,6.6′-tetrachloro-1.1′,3.3′-tetraethylbenzimidazolyl-carbocyanine iodide (JC-1) was employed to assess the damage to the polarization of the mitochondrial membrane potential (MMP) by CLSM. The results demonstrated that CMS@B@I moderated red fluorescence and increased green fluorescence, as opposed to control and H_2_O_2_ groups (Figure 3H and Supplementary Figure S8). This implies the polarization of MMP and the apoptosis of tumor cells. As predicted, the cellular green fluorescence intensity was further increased after NIR-II irradiation, consistent with ROS results. The above results demonstrate that CMS@B@I can induce an ROS storm, owing to the photothermal-enhanced Fenton activity and PDT effect, thus resulting in cell death via increasing cellular oxidative stress.

Prior to the treatment in mice, fluorescence and infrared thermal imaging were performed to trace the fate of CMS@B@I. All animal protocols were approved by the Ethical Committee of Fujian Medical University. NIR-II Fluorescence was detected before and after tail vein administration for 1, 3, 5,7, 9, 12, and 24 h. The mice were exposed to an 808 nm laser. According to Figure 4A and Supplementary Material, the fluorescence signal in the tumor site gradually increased. The results demonstrated that CMS@B@I has excellent tumor accumulation via the enhanced permeability and retention (EPR) effect (Yuan et al., 1995). Additionally, thermal imaging was performed upon NIR-II laser irradiation after tail vein injection for 24 h. As indicated in Figure 4B, the temperature of the tumor site was rapidly increased, as opposed to PBS group, confirming the excellent PTT capacity of CMS@B@I. These imaging results demonstrated that CMS@B@I is a bimodal imaging-guided and promising CDT nano-agent for PDT and photothermal-enhanced catalytic therapy.

**FIGURE 5:**
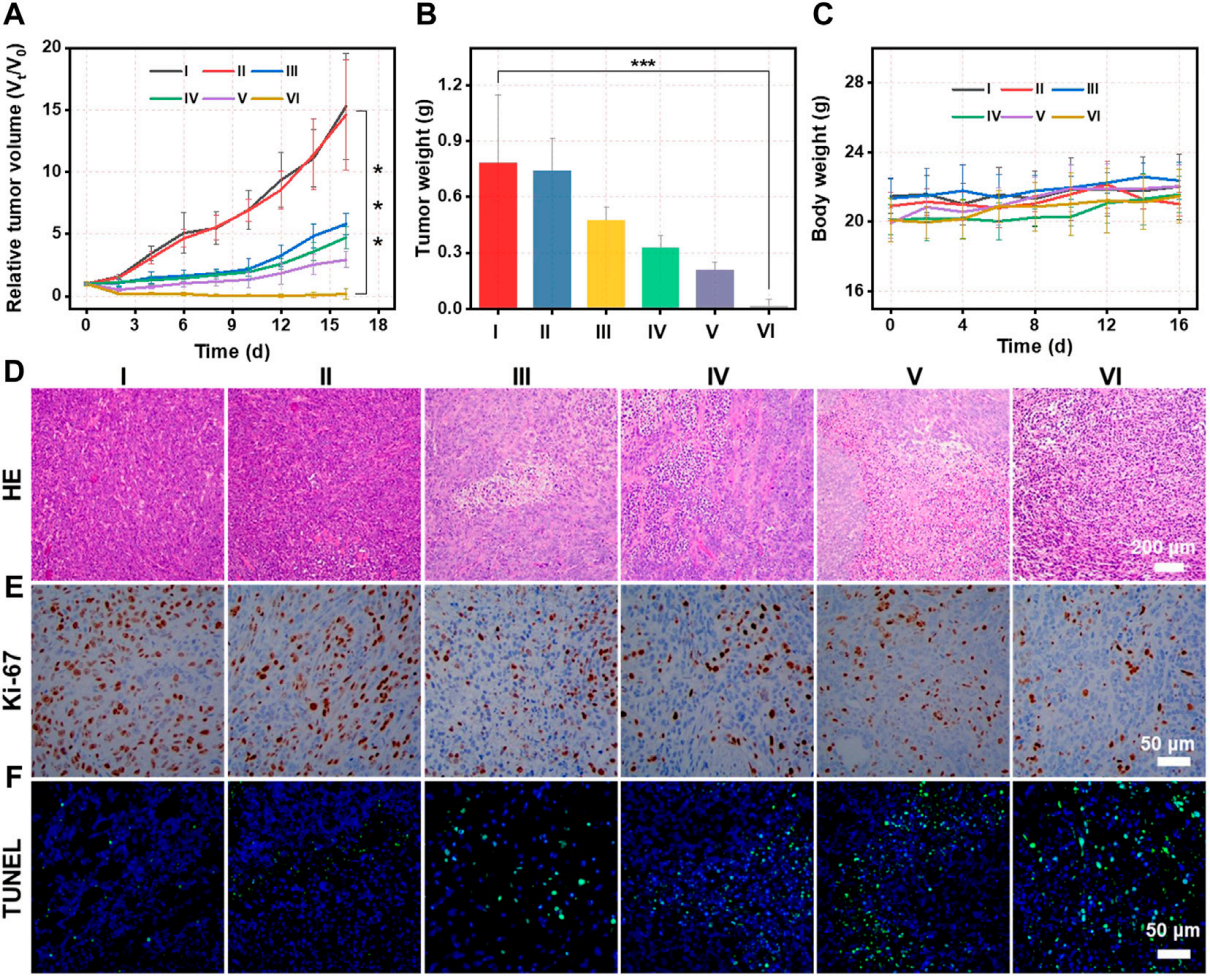
**(A)** The tumor growth curves of mice treated with different formulations. I (PBS), II (PBS plus L), III (CMS@B@I), IV (CMS@B@I plus 808 nm laser), V (CMS@B@I plus 1,064 nm laser), and VI (CMS@B@I plus 808 nm and 1,064 nm lasers). **(B)** The average weights of tumor dissected from mice. **(C)** The fluctuation of body weight after different treatments. **(D)** H&E staining, **(E)** Ki-67 and **(F)** TUNEL of tumor slides from different group. *p < 0.05, **p < 0.01, ***p < 0.001.

Inspired by the optimal PDT effect and photothermal-enhanced Fenton activity of CMS@B@I *in vitro*, *in vivo* therapeutic efficacy was carried out on 4T1 tumor-bearing mice after intravenous (i.v.) administration. The 4T1 tumor-bearing mice were randomly divided into six groups, with five mice in each group: PBS (group I), PBS plus laser (group II), CMS@B@I (group III), CMS@B@I plus 808 nm laser (group IV), CMS@B@I plus 1,064 nm laser (group V), and CMS@B@I plus 808 and 1,064 nm lasers (group VI), when the tumor volume reached approximately 100 mm^3^. CMS@B@I was administered once via i. v. Injection, followed by 808 nm or 1,064 nm laser (1 mW/cm^2^) exposure for 5 min, 24 h after the injection. The body weight and the tumor volume of all mice were measured every other day. The curves of the tumor growth indicate that CMS@B@I alone can effectively inhibit tumor growth, as opposed to the PBS group, since CMS@B@I produced abundant ROS in tumor sites (Figure 5A and Supplementary Figure S10). As predicted, the results demonstrate that CMS@B@I can inhibit tumor growth. The inhibition of tumor growth was further remarkably enhanced after 808 nm laser irradiation, indicating the combination of CDT and PDT effects. More importantly, the tumor growth was further suppressed after being exposed to 1,064 nm irradiation, indicating the photothermal-enhanced catalytic activity of CMS@B@I. The average tumor weight results were consistent with the tumor growth curves (Figure 5B). Additionally, no explicit variation in body weight was observed within the evaluation period, indicating the excellent biocompatibility of CMS@B@I (Figure 5C). Furthermore, the tumors were collected to investigate the proliferation and cell death level by hematoxylin and eosin (H&E) staining, Ki-67 staining and the terminal deoxynucleotidyl transferase-mediated dUTP nick-end labeling (TUNEL)-staining. H&E and TUNEL results demonstrate that the CMS@B@I group exhibited significant damage of the tumor. This indicates the amplified cellular oxidative stress via producing sufficient ROS (Figures 5D,E). The most serious damage was induced by a combination with NIR-II irradiation, confirming photothermal-enhanced catalytic therapy. Furthermore, Ki-67 staining demonstrated explicit inhibition of tumor proliferation, in agreement with H&E staining (Figure 5F). H&E staining of major organs indicated no obvious change compared to the control group (Supplementary Figure S11). These results demonstrate that CMS@B@I can effectively inhibit tumor growth with negligible side effects via PDT and photothermal-enhanced CDT effects.

## Conclusion

In this study, a bimodal imaging-guided CDT nanoagent (CMS@B@I) was designed, which achieved superior PDT and photothermal-enhanced CDT effects. CMS@B@I not only exhibited excellent Fenton activity, owing to its multivalent elements of Cu and Mn, but also produced sufficient ROS after exposure to 808 nm laser irradiation, amplifying cellular oxidative stress. Additionally, CMS@B@I possessed superior photothermal performance (PCE = 35.3%) under 1,064 nm laser irradiation, and was able to significantly enhance the catalytic activity of tumor therapy. Both *in vitro* and *in vivo* experimental results demonstrated that CMS@B@I not only performed bimodal imaging (NIR-II fluorescence and infrared thermal imaging) on tracing the therapeutic effect, but also remarkably inhibited tumor growth with negligible side effects. This study provides an innovative strategy to fabricate imaging-guided and photothermal-enhanced CDT nanoagents for tumor catalytic therapy, which highlights its promising clinical potential.

## Data Availability

The raw data supporting the conclusions of this article will be made available by the authors, without undue reservation.
