# Supplementary material for: Biomineralized hybrid nanodots for tumor therapy via NIR-II fluorescence and photothermal imaging
Source: Front Bioeng Biotechnol. 2022 Oct 31;10:1052014. doi: 10.3389/fbioe.2022.1052014 (PMC9660244; doi:10.3389/fbioe.2022.1052014)
Supplement: Supplementary file 1 [file DataSheet1.docx]

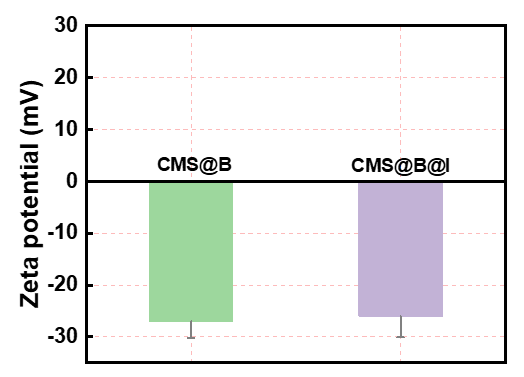


**Figure S1**. The zeta potentials of different nanoparticles.


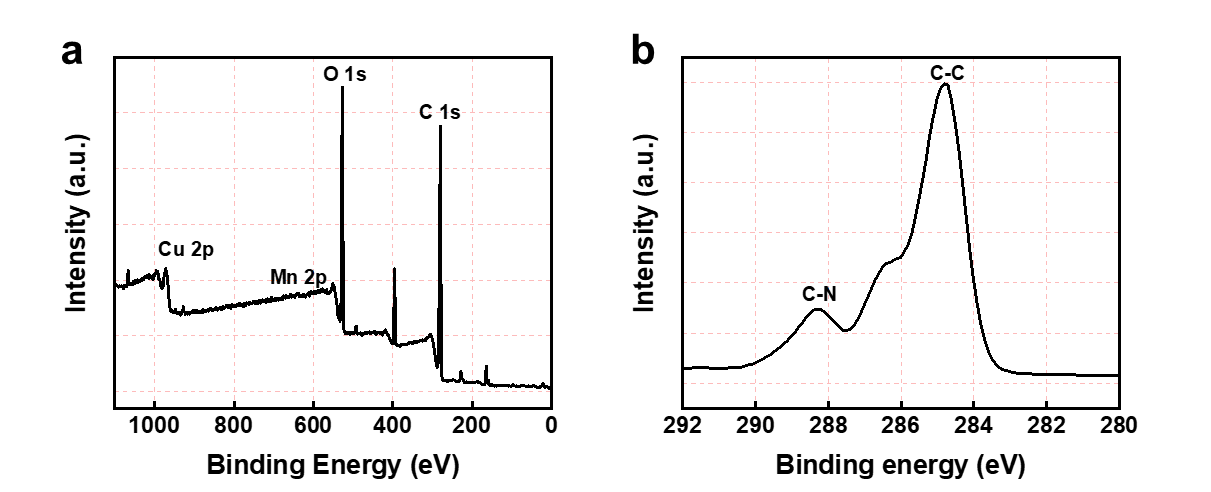


**Figure S2**. (a) XPS survey spectrum and (b) high resolution C 1s XPS spectrum of CMS@B@I.

**Figure S3.** The stability of CMS@B@I in DMEM.


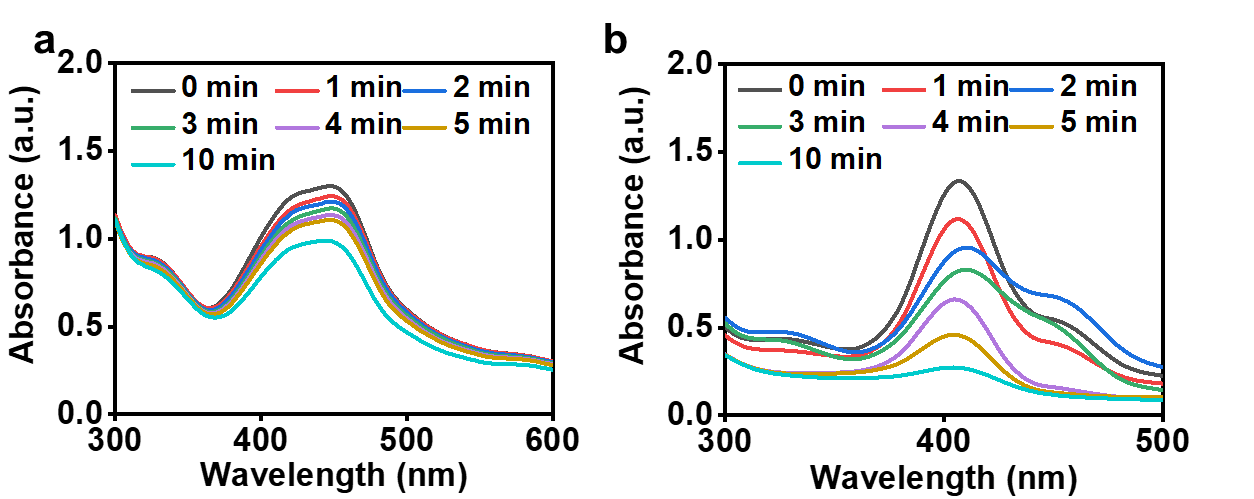


**Figure S4**. The detection of hydroxyl radical by using DPBF kit.


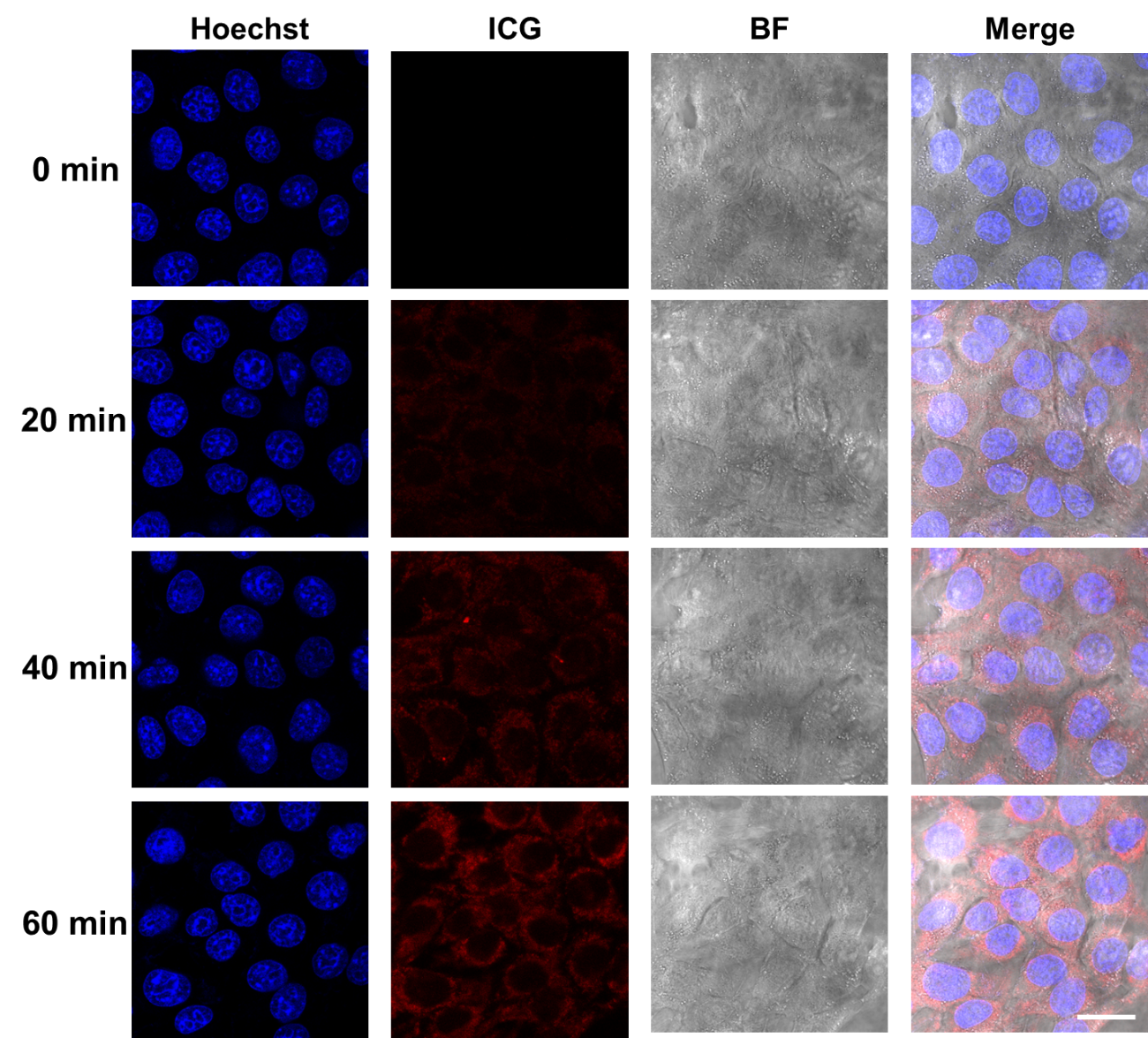


**Figure S5**. The cellular uptake behavior of CMS@B@I.


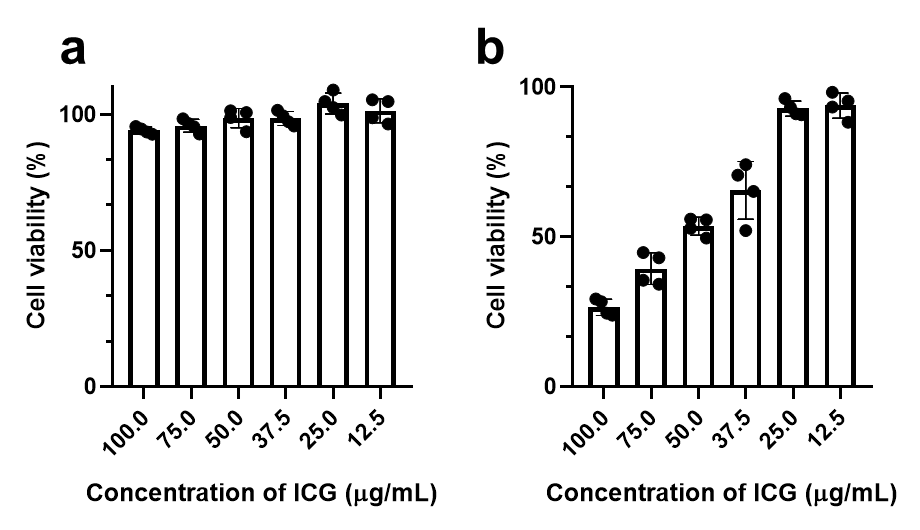


**Figure S6**. The cell viability of 4T1 cells after 24 h treatment with ICG and ICG plus laser.


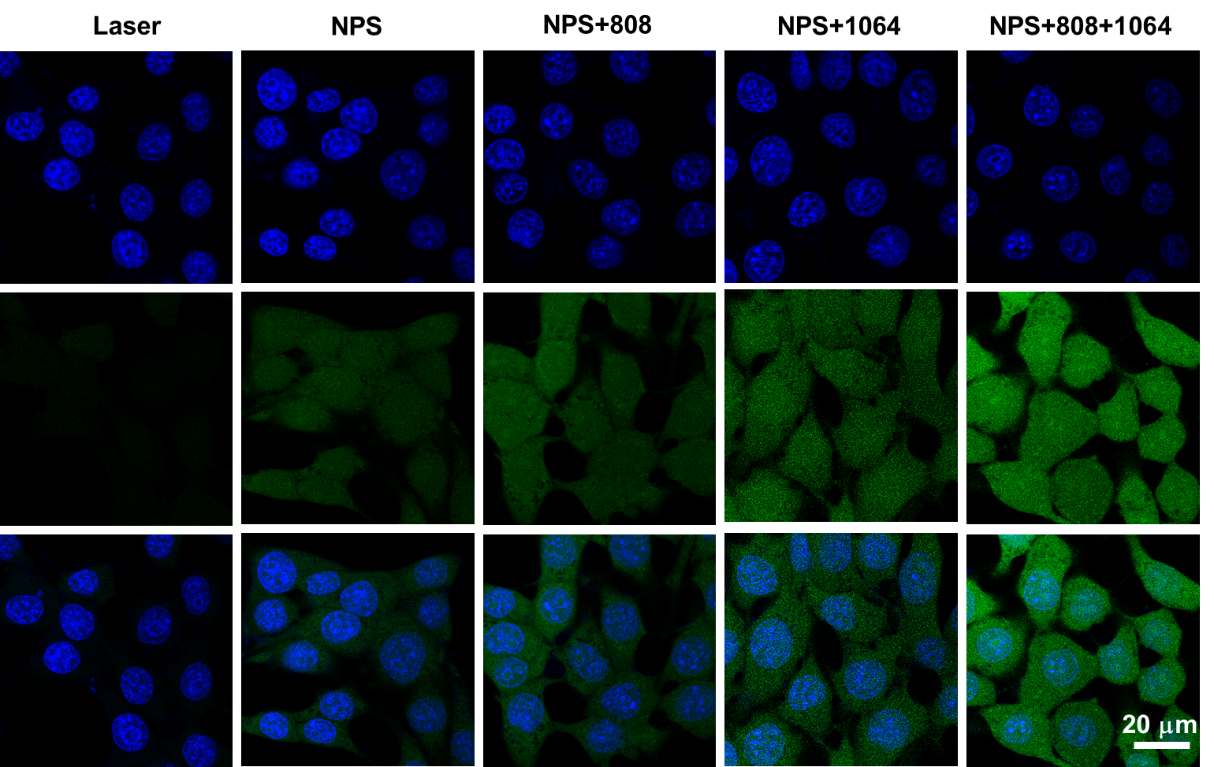


**Figure S7**. The ROS level in 4T1 cells treated with various formulations.

**
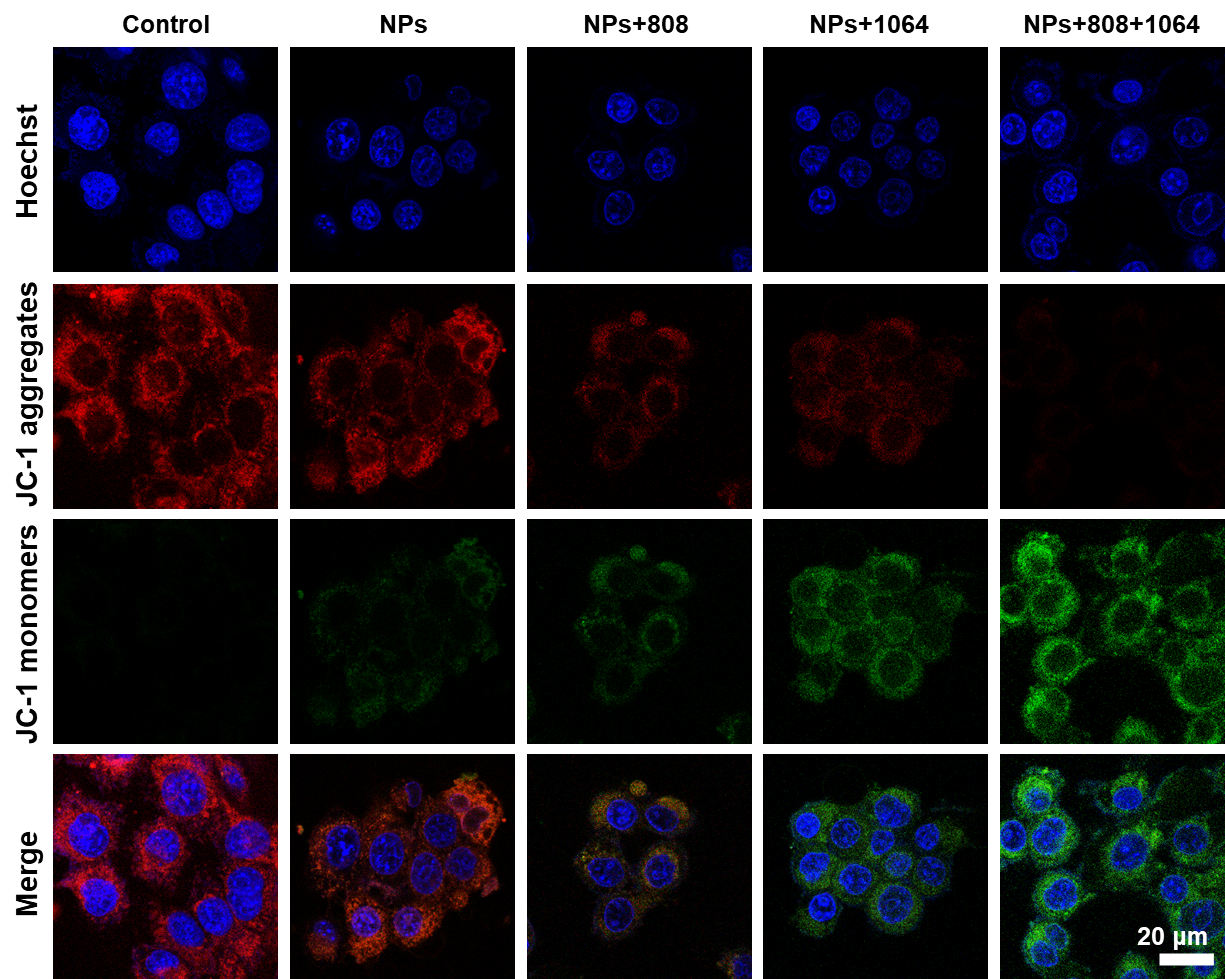
**

**Figure S8**. Fluorescence images of mitochondrial membrane potential (JC-1 staining) in 4T1-7 cells treated with various formulations.


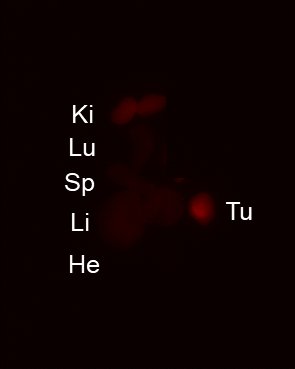


**Figure S9**. *Ex vivo* fluorescence image of major organs and tumor at 24 h post-injection of CMS@B@I. Ki (kidney), Lu (Lung), Sp (spleen), Li (liver), He (Heart), and Tu (tumor).


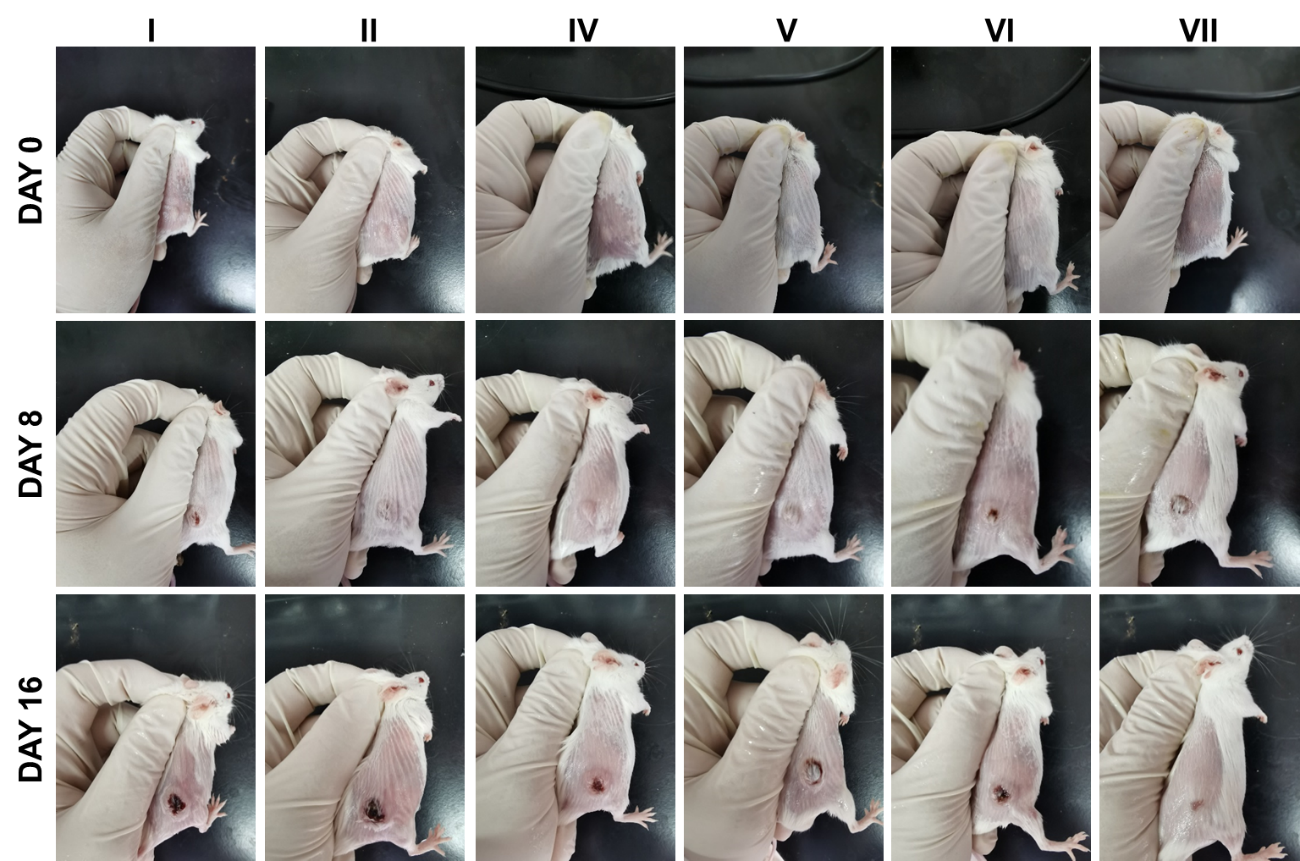


**Figure S10**. The photographs of mice treated with different formulations at the end of treatment. I (PBS), II (PBS plus L), III (CMS@B@I), IV (CMS@B@I plus 808 nm laser), V (CMS@B@I plus 1064 nm laser), and VI (CMS@B@I plus 808 nm and 1064 nm lasers).

**Figure S11**. H&E staining of major organs with different treatments. I (PBS), II (PBS plus L), III (CMS@B@I), IV (CMS@B@I plus 808 nm laser), V (CMS@B@I plus 1064 nm laser), and VI (CMS@B@I plus 808 nm and 1064 nm lasers).
